# Supplementary figures and images for: ERK3/MAPK6 controls IL-8 production and chemotaxis
Source: eLife. 2020 Apr 21;9:e52511. doi: 10.7554/eLife.52511 (PMC7192585; doi:10.7554/eLife.52511)

Figure 1

C

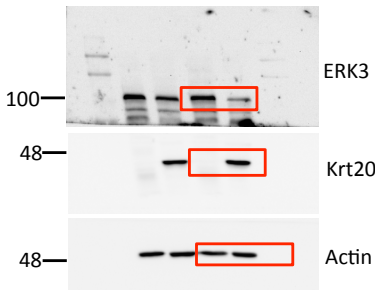

F

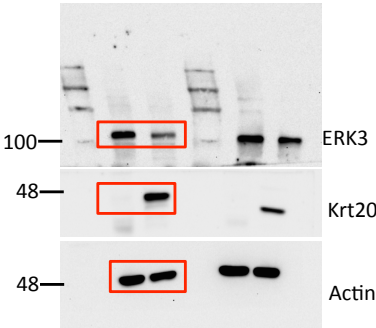

G

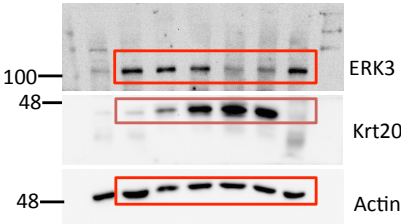

Supplement: Figure 1—source data 1. [file elife-52511-fig1-data1.pdf]

Figure 2

A

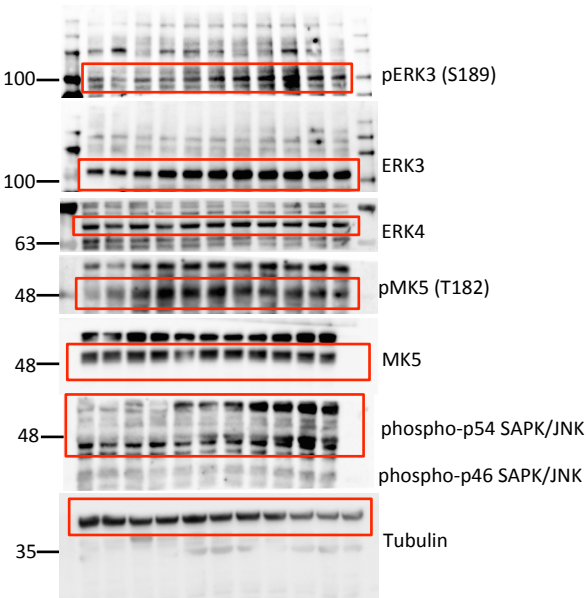

E

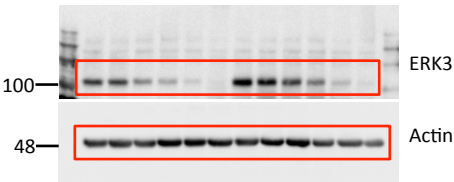

G

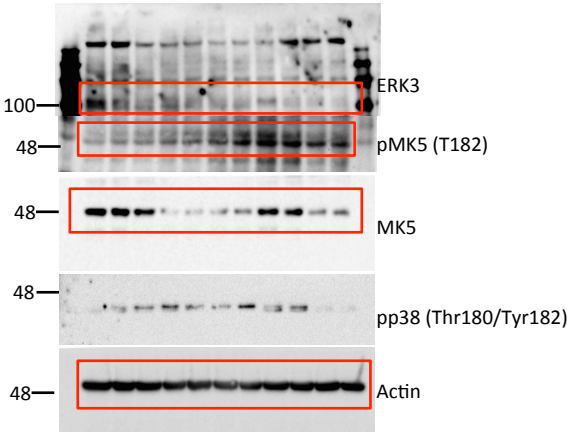

M

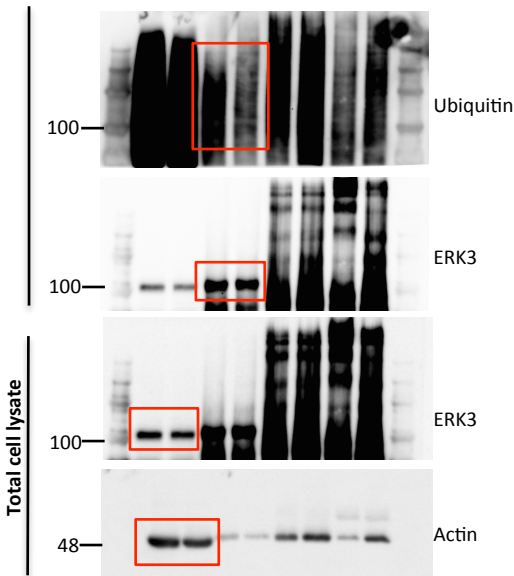

N

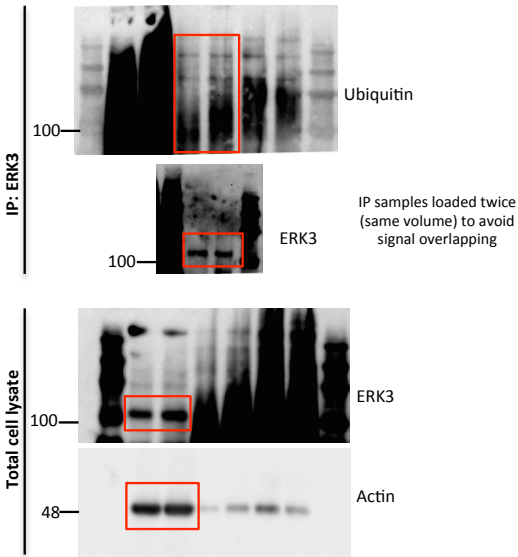

Supplement: Figure 2—source data 1. [file elife-52511-fig2-data1.pdf]

Figure 2-figure supplement 1

A

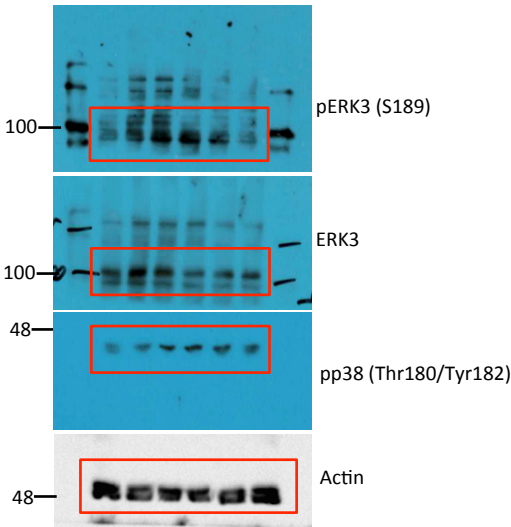

Supplement: Figure 2—figure supplement 1—source data 1. [file elife-52511-fig2-figsupp1-data1.pdf]

Figure 2-figure supplement 2

**A**

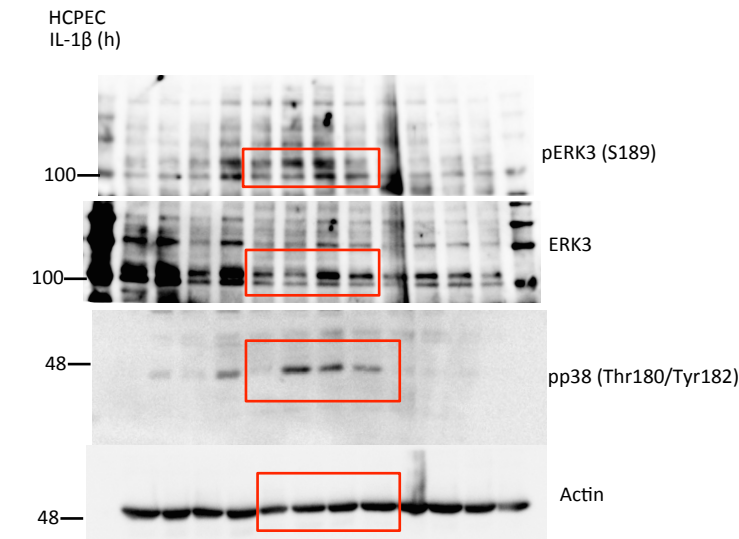

**B**

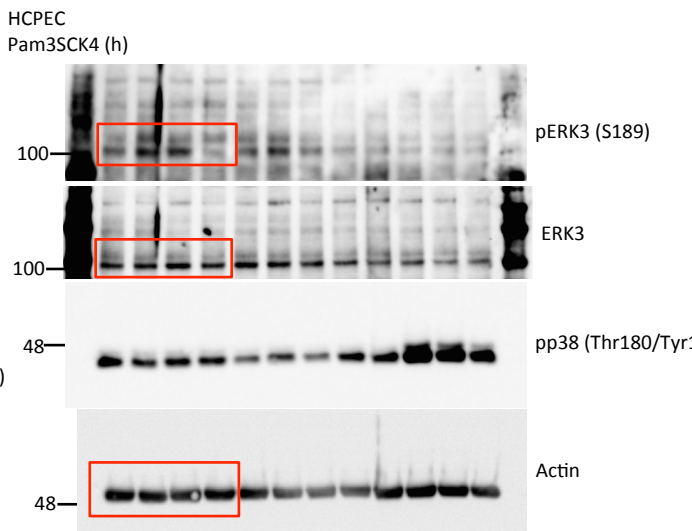

**C**

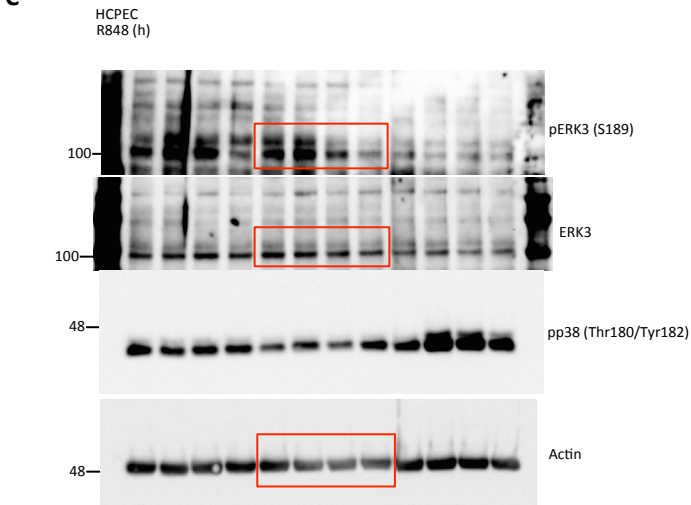

**D**

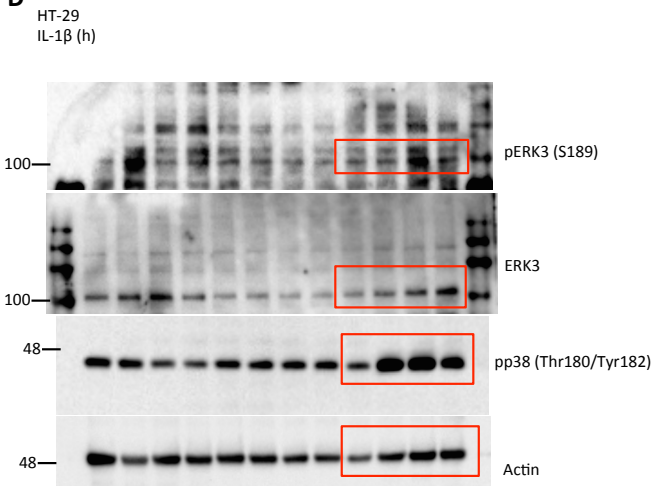

**E**

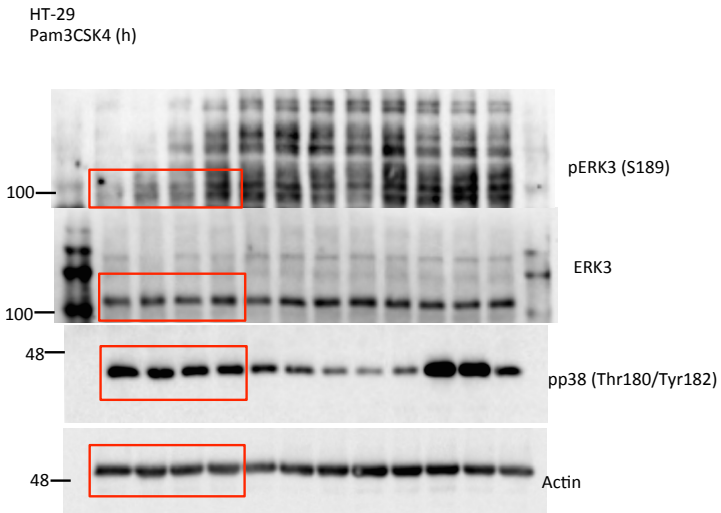

**F**

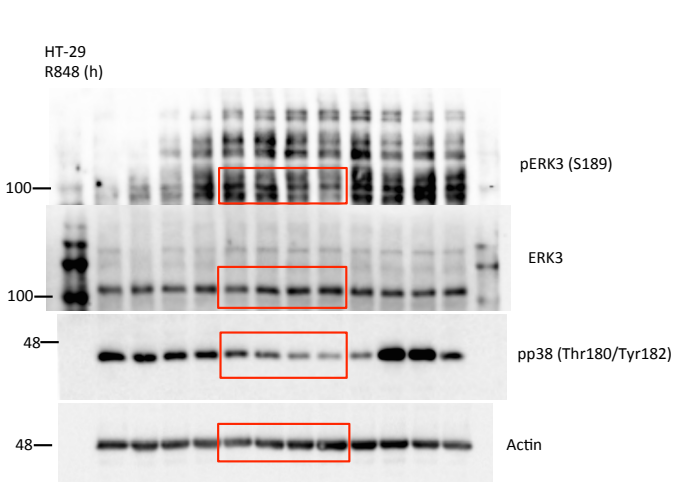

Supplement: Figure 2—figure supplement 2—source data 1. [file elife-52511-fig2-figsupp2-data1.pdf]

Figure 3

A

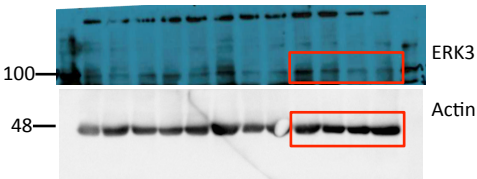

Supplement: Figure 3—source data 4. [file elife-52511-fig3-data4.pdf]

Figure 4

D

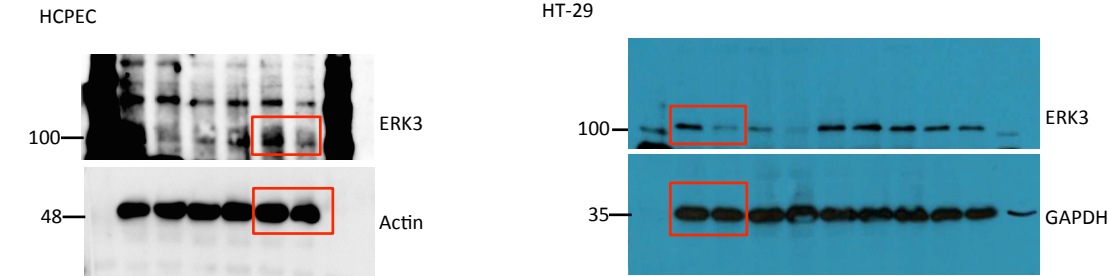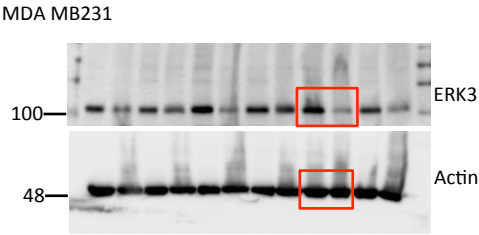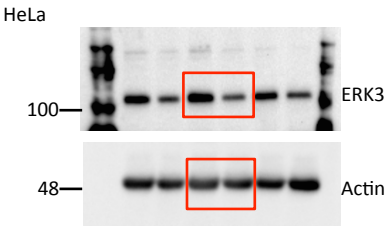

G

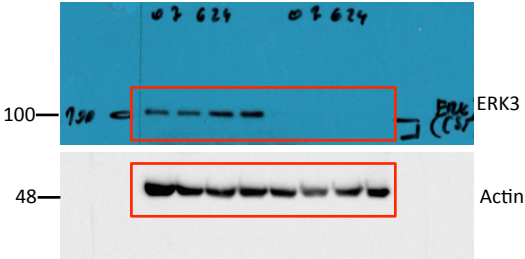

J

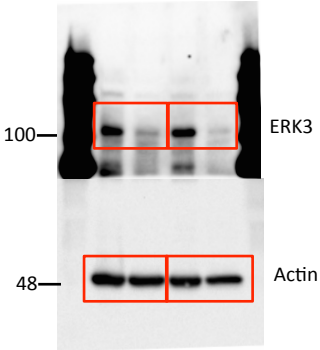

Supplement: Figure 4—source data 1. [file elife-52511-fig4-data1.pdf]

Figure 4-figure supplement 2

A

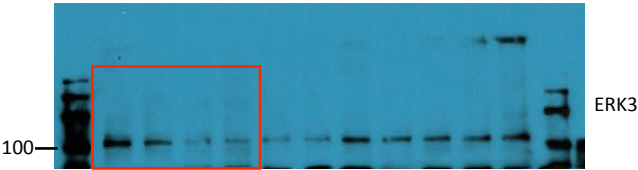

B

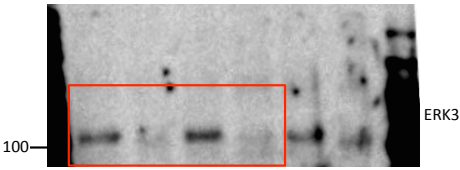

Supplement: Figure 4—figure supplement 2—source data 1. [file elife-52511-fig4-figsupp2-data1.pdf]

Figure 4-figure supplement 3

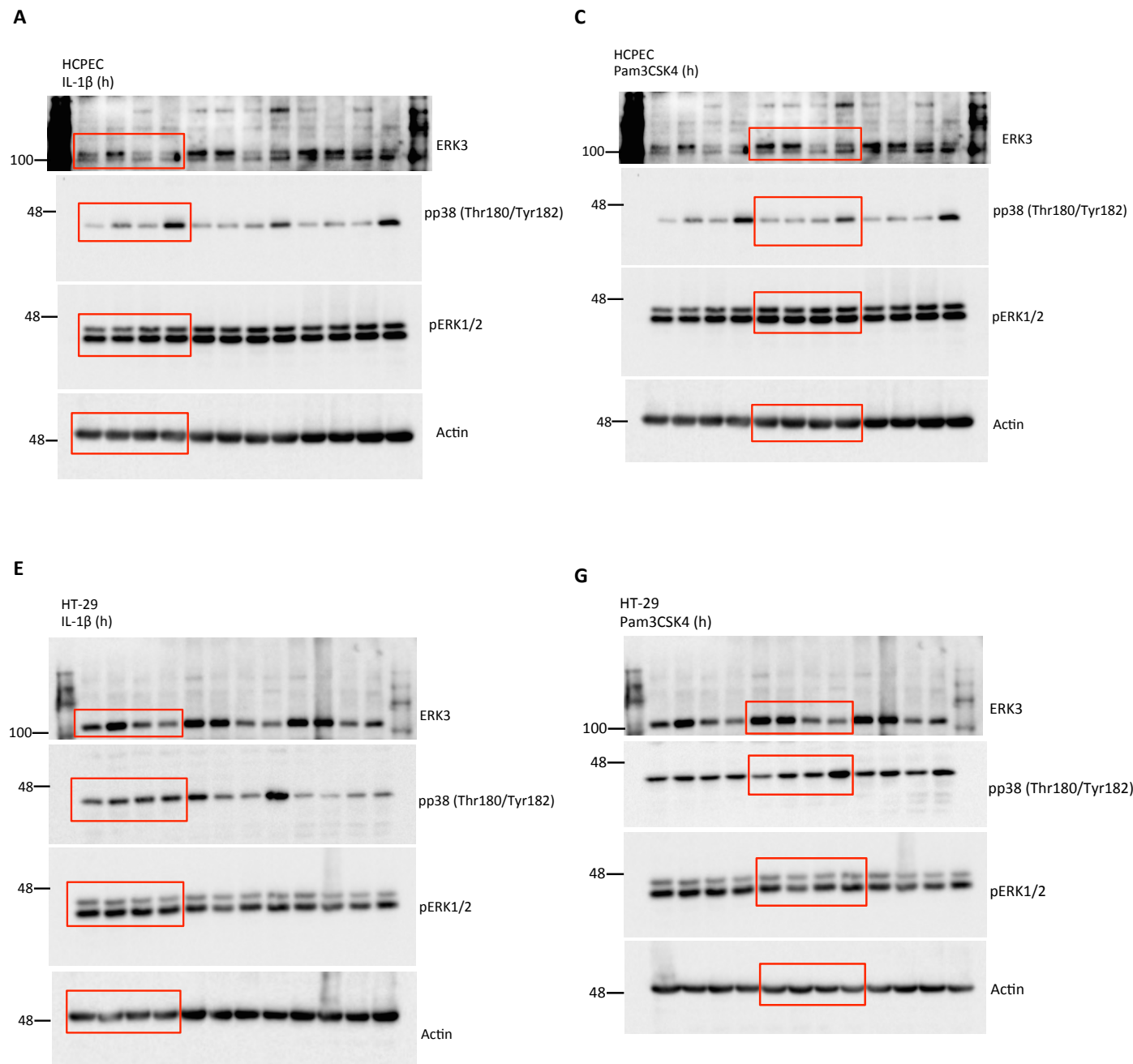

Supplement: Figure 4—figure supplement 3—source data 1. [file elife-52511-fig4-figsupp3-data1.pdf]

Figure 4-figure supplement 4

A

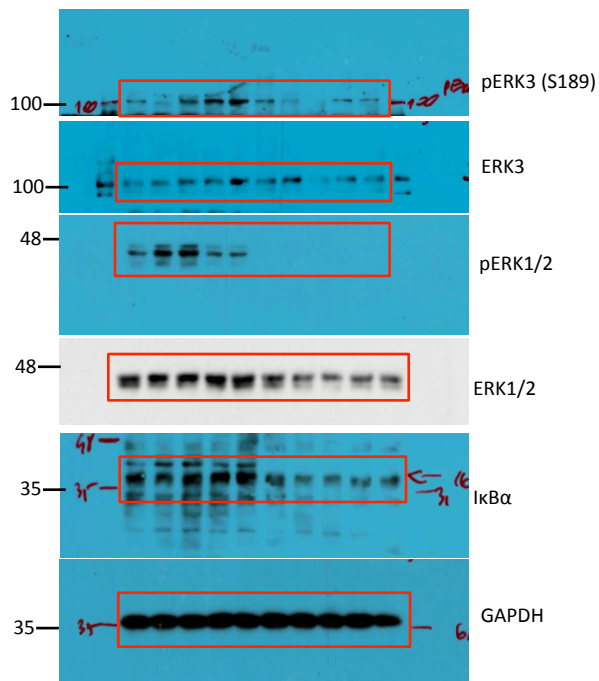

B

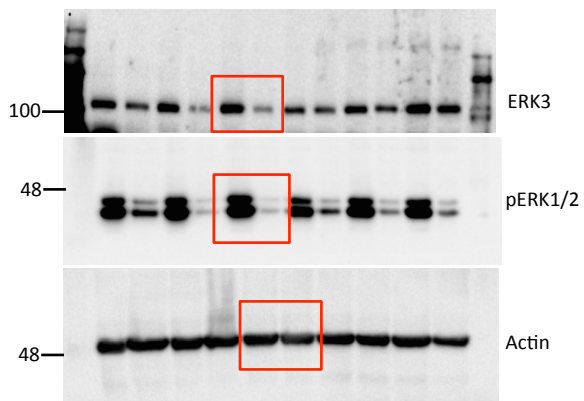

D

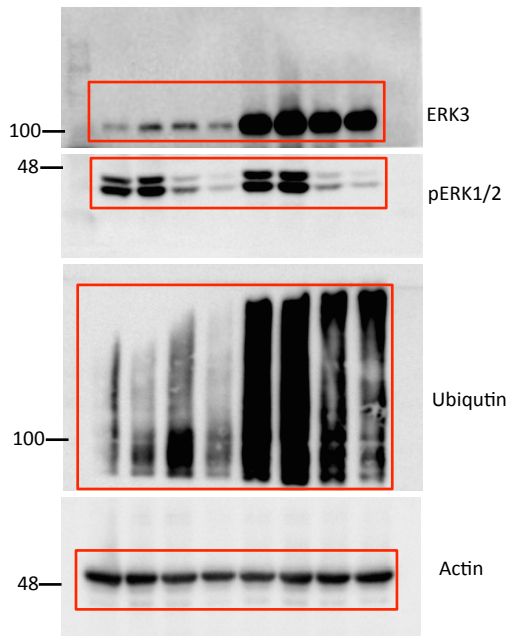

Supplement: Figure 4—figure supplement 4—source data 1. [file elife-52511-fig4-figsupp4-data1.pdf]

Figure 4-figure supplement 5

**A**

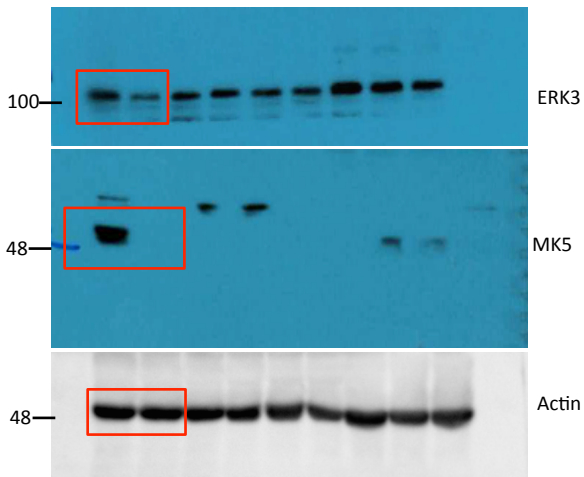

**C**

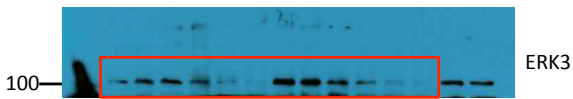

Supplement: Figure 4—figure supplement 5—source data 1. [file elife-52511-fig4-figsupp5-data1.pdf]

Figure 5

A

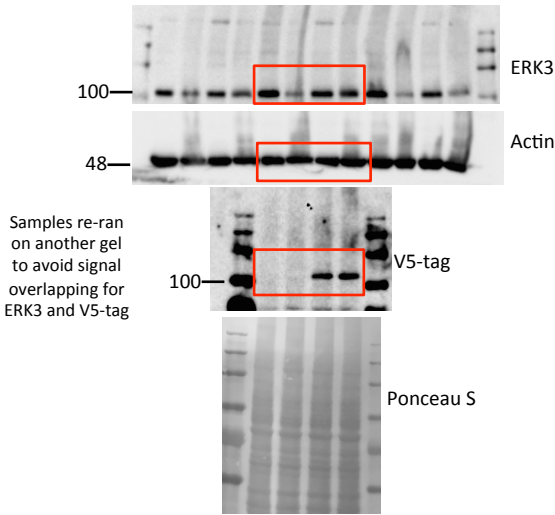

B

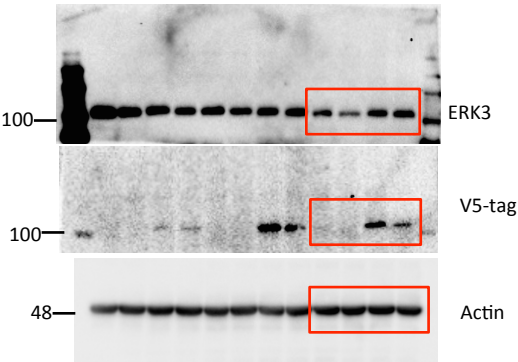

C

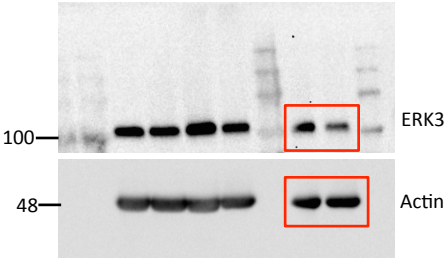

Supplement: Figure 5—source data 1. [file elife-52511-fig5-data1.pdf]

Figure 6

A

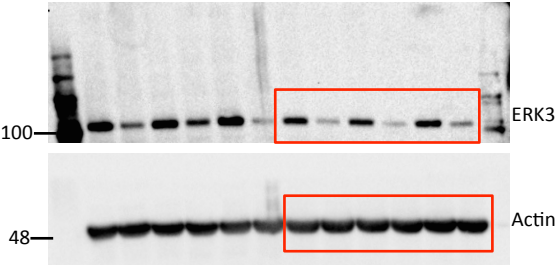

Supplement: Figure 6—source data 2. — Table represents activity of TF analyzed in control and ERK3-depleted HCPECs. [file elife-52511-fig6-data2.pdf]

Figure 6-figure supplement 3

**B**

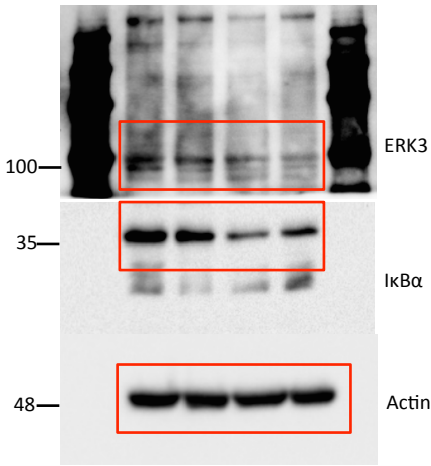

Supplement: Figure 6—figure supplement 3—source data 1. [file elife-52511-fig6-figsupp3-data1.pdf]

Figure 7

A

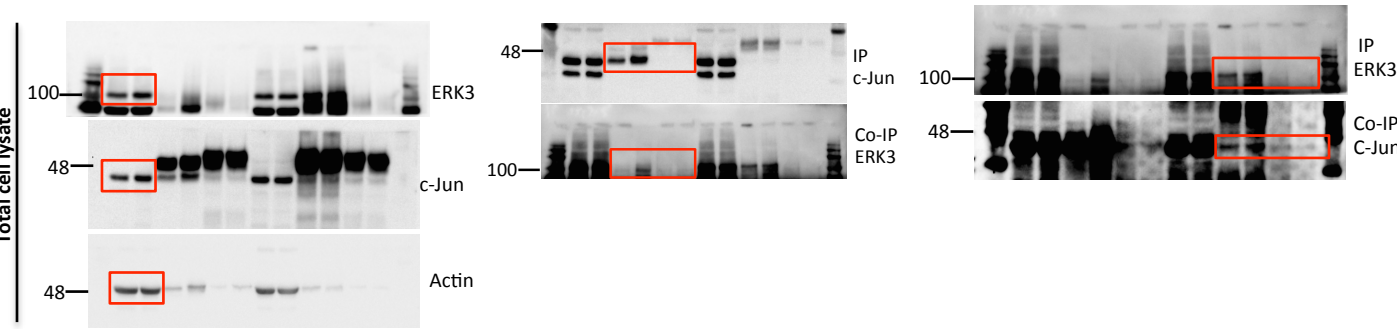

C

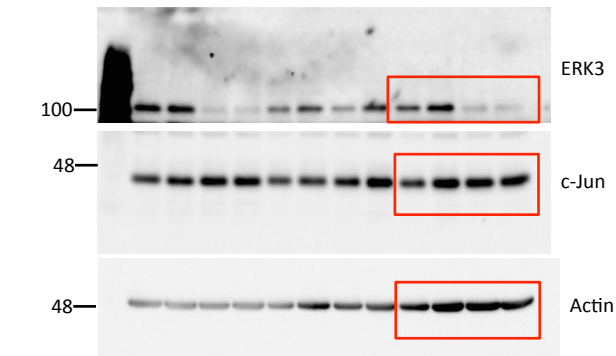

Supplement: Figure 7—source data 1. [file elife-52511-fig7-data1.pdf]

Figure 7-figure supplement 1

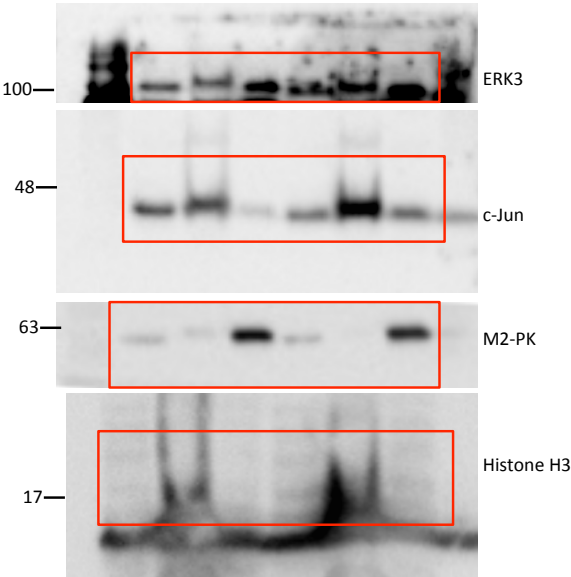

Supplement: Figure 7—figure supplement 1—source data 1. [file elife-52511-fig7-figsupp1-data1.pdf]
